# Fewer acute respiratory infection episodes among patients receiving treatment for gastroesophageal reflux disease

**DOI:** 10.1371/journal.pone.0172436

**Published:** 2017-02-21

**Authors:** Herng-Ching Lin, Sudha Xirasagar, Shiu-Dong Chung, Chung-Chien Huang, Ming-Chieh Tsai, Chao-Hung Chen

**Affiliations:** 1 School of Health Care Administration, Taipei Medical University, Taipei, Taiwan; 2 Research Center of Sleep Medicine, College of Medicine, Taipei Medical University, Taipei, Taiwan; 3 Department of Health Services Policy and Management, Arnold School of Public Health, University of South Carolina, Columbia, United States of America; 4 Department of Surgery, Far Eastern Memorial Hospital, Banciao, Taipei, Taiwan; 5 Division of Gastroenterology, Department of Internal Medicine, Cathay General Hospital, Hsinchu Branch, Taiwan; 6 Department & Institute of Physiology, National Yang-Ming University, Taipei, Taiwan; 7 Department of Cosmetic Applications and Management, Mackay Junior College of Medicine, Nursing, and Management, Taipei, Taiwan; 8 Department of Thoracic Surgery, MacKay Memorial Hospital, Taipei, Taiwan; University Hospital Llandough, UNITED KINGDOM

## Abstract

Patients with gastroesophageal reflux disease (GERD) present with comorbid complications with implications for healthcare utilization. To date, little is known about the effects of GERD treatment with a proton-pump inhibitor (PPI) on patients’ subsequent healthcare utilization for acute respiratory infections (ARIs). This population-based study compared ARI episodes captured through outpatient visits, one year before and one year after GERD patients received PPI treatment. We used retrospective data from the Longitudinal Health Insurance Database 2005 in Taiwan, comparing 21,486 patients diagnosed with GERD from 2010 to 2012 with 21,486 age-sex matched comparison patients without GERD. Annual ARI episodes represented by ambulatory care visits for ARI (visits during a 7-day period bundled into one episode), were compared between the patient groups during the 1-year period before and after the index date (date of GERD diagnosis for study patients, first ambulatory visit in the same year for their matched comparison counterpart). Multiple regression analysis using a difference-in-difference approach was performed to estimate the adjusted association between GERD treatment and the subsequent annual ARI rate. We found that the mean annual ARI episode rate among GERD patients reduced by 11.4%, from 4.39 before PPI treatment, to 3.89 following treatment (mean change = -0.5 visit, 95% confidence interval (CI) = (-0.64, -0.36)). In Poisson regression analysis, GERD treatment showed an independent association with the annual ARI rate, showing a negative estimate (with *p*<0.001). The study suggests that GERD treatment with PPIs may help reduce healthcare visits for ARIs, highlighting the importance of treatment-seeking by GERD patients and compliance with treatment.

## Introduction

Gastroesophageal reflux disease (GERD), which often presents with symptoms of heartburn and acid regurgitation, is a common chronic disorder in many countries [1]. In Western populations, about 10%~20% of the population report having GERD symptoms at least weekly [2,3], while in East Asia, the prevalence ranges between 2.5%~7.8% [2,4,5]. A rising trend of GERD prevalence is documented in population-based surveys, potentially attributable to aging populations, the obesity epidemic and the associated dietary and physical activity changes, and changes in sleep patterns [6,7]. GERD is documented to markedly impair general well-being, quality of life, and work productivity, in addition to requiring substantial medical care resources [2,8].

The most overt complications of GERD are esophageal, primarily due to injury. The complications include erosive esophagitis with or without acute or chronic gastrointestinal hemorrhage, esophageal stricture, and Barrett’s esophagus [9]. In addition, patients with GERD frequently report extra-esophageal symptoms ranging from pharyngitis, laryngitis, asthma, and hoarseness to more serious pulmonary aspiration syndromes, including bronchiectasis, lung abscesses, and recurrent pneumonias [10,11]. The airways, in contrast to the distal esophagus, are not protected by antireflux clearance mechanisms or intrinsic mucosal properties. One prospective study of patients categorized into GERD and non-GERD patients by endoscopy findings, reported a strong relationship between gastroesophageal reflux symptoms and various respiratory disorders [12]. Among patients with erosive esophagitis, respiratory symptoms were more prevalent, with evidence of a direct association between the severity of airway obstruction and that of GERD symptoms.

GERD patients, presenting with esophageal or extra-esophageal symptoms, have more comorbid complications that require more healthcare visits, thus incurring higher healthcare costs [13]. Anti-secretory agents, such as proton pump inhibitors (PPIs) provide both symptom relief and healing of short- and long-term erosive esophagitis [14]. Few studies have examined whether GERD treatment ameliorates the non-esophageal complications and co-morbidities. Littner et al. reported that medical treatment of acid reflux disease had no significant effects on daily asthma symptoms or pulmonary function [15]. To date, little is known about whether treating GERD patients with a PPI affects subsequent occurrences of acute respiratory infections or healthcare utilization for these episodes. This population-based study used nation-wide longitudinal data to examine changes in the annual acute respiratory infection (ARIs) episode rate identified through ambulatory care visits, one year before and one year after adult GERD patients received PPI treatment.

## Methods

### Database

Data for this study were retrieved from the Longitudinal Health Insurance Database 2005 (LHID2005). The LHID2005 includes registration files and filed medical claims for 1,000,000 enrollees randomly selected from all enrollees listed in the 2005 Registry of Beneficiaries under the Taiwan National Health Insurance (NHI) program (*n* = 23.72 million). Specifically, in 1995, Taiwan initiated its NHI program to grant universal coverage to all citizens, with a generous benefit package, low co-payments, and a free choice of widely-dispersed networks of healthcare providers. Monthly claims summaries in the claims dataset consist of outpatient and inpatient claims for every NHI beneficiary, details of inpatient and drug orders, the contracted medical facility used, and board-certified specialists who attended the patient. While the diagnostic coding accuracy cannot be ascertained, claims accuracy is considered to be generally accurate due to the NHI Bureau’s routine practice of sample verification of each hospital’s claims using medical charts, followed by punitive measures for coding infractions. Many researchers have carried out longitudinal studies using the LHID2005 data on the 1,000,000 representative sample of NHI enrollees and published study results in internationally peer-reviewed journals. The LHID2005 provides a unique opportunity to study the relationship between treating GERD and subsequent healthcare service utilization for ARIs. Because the study dataset consists of de-identified secondary data released to the researcher community for research purposes, the study was exempted from full IRB review by the Taipei Medical University Institutional Review Board (TMU-JIRB 201412035).

### Study sample

The study included a study group and a comparison group. To identify the study group, we first selected 33,730 patients with a first-time principal diagnosis of GERD (ICD-9-CM code 530.11 or 530.81) between 1^st^ January 2010 and 31^st^ December 2012 in the LHID2005. We excluded 559 patients aged <18 years, 162 patients who died during the following year, and 11,523 patients who did not have endoscopy-confirmed GERD diagnosis or those with endoscopy-confirmed diagnosis who had not received a prescription PPI for at least 30 days following diagnosis. In Taiwan, eligibility for NHI reimbursement for PPI prescriptions for GERD requires completion of a peer review process, conducted by a gastroenterologist sub-committee in the NHI Administration. The committee assesses the patient’s eligibility for PPIs based on clinical symptoms and the endoscopy imaging evidence, applying the Los Angeles Classification of Esophagitis criteria for a GERD diagnosis. Our final analytic sample of study patients consisted of 21,486 GERD patients, and we assigned their first ambulatory care visit with a PPI prescription for GERD as their index date.

We selected comparison patients from the remaining LHID2005 enrollees aged ≥ 18 years using the SAS proc survey select program for comparison patient selection (SAS System for Windows, vers. 8.2, SAS Institute, Cary, NC). We excluded all enrollees with a GERD diagnosis during the study years, and those who died during the year 2013 (to ensure equal follow-up periods (1 year). We then randomly selected 21,486 patients matched with the study patients on sex, age group (18~29, 30~39, 40~49, 50~59, 60~69, and >69 years), and year of index date of the corresponding study patient. For the study group, the year of the index date was defined as the year in which the study group was assigned their first ambulatory visit for GERD treatment. Comparison patients were selected based on a match of their year of utilization of any ambulatory care with the year of GERD treatment of a study patient (of the same sex and age group). For the comparison patients, we defined the date of their first healthcare use in the index year as their index date.

The final study sample consists of 42,972 patients. We calculated the mean number of ambulatory care visits (including all outpatient visits to hospitals and clinics) for ARIs (ICD-9-CM codes 460~466) for each patient within a 1-year period before and after their index date, and bundled all ARI visits within 7 days into one episode (to avoid multiple counting of a single ARI episode). Although most ARIs are self-limited viral infections, medications such as antibiotics may be prescribed for the management of ARIs.

### Statistical analysis

**Fig 1:**
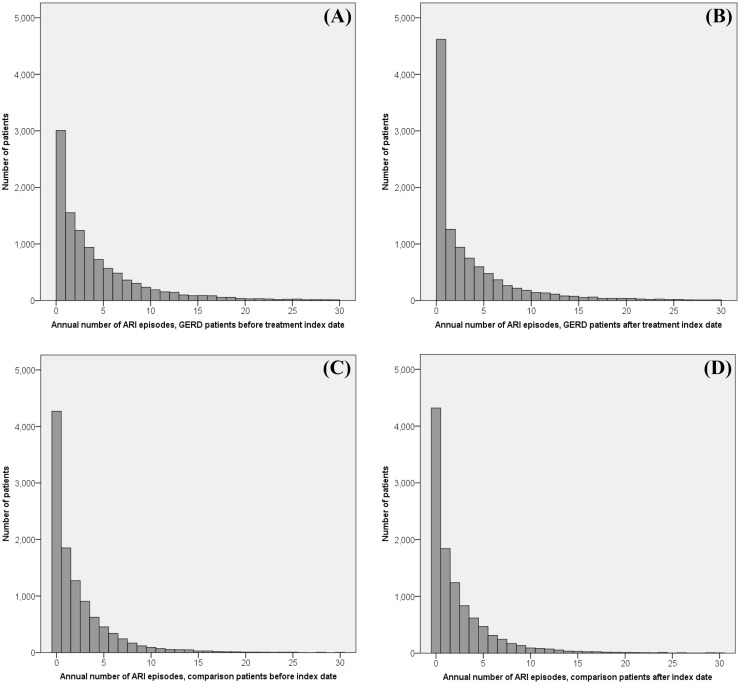
Distribution of patients by annual number of for acute respiratory infection episodes classified by patient group and study period: (A) GERD patients before the index date (B) GERD patients after the index date (C) Comparison patients before the index date (D) Comparison patients after the index date.

We used the SAS statistical package (SAS System for Windows, vers. 8.2) and SPSS statistical software (IBM SPSS Statistics for Windows, Version 20.0.) for analyses. We used paired *t*-tests to compare mean annual ambulatory ARI episodes during the year before and after the index date. We used the difference-in-difference method to compare the average change over time in the annual ARI episode rate for the GERD group, and further, compared the before-after change in the GERD-treated group with that of the comparison group. Based on the distribution of ARI episodes in the sample (Fig 1), we conducted Poisson regression analysis, using the difference-in-difference method to estimate the independent association between GERD treatment and annual ARI episode rate, adjusted for age, sex, monthly income, geographic region, and residential urbanization level. The difference-in-difference estimate of the impact of GERD treatment on the ARI episode rate was assessed by the estimate for the interaction term between GERD treatment (yes vs. no) and time period (before vs. after). We had no missing data on the sociodemographic covariates, as these are required claims form items to qualify for reimbursement. Differences were considered significant based on a two-sided *p* value of 0.05.

## Results

The sample mean age was 52.9 years (±15.7) for the total sample, 53.0±15.4 years for study group, and 52.9±15.9 years for the comparison group. Table 1 shows that after matching for sex, age group, and the index year, chi-square tests showed slight, though statistically significant differences in monthly income, geographic region, and residential urbanization level between the GERD sample and comparison sample (all *p<*0.001). The GERD group had somewhat more urbanized patients and had slightly higher income than the non-GERD group.

**Table 1:** Demographic characteristics of sample patients (N = 42,972).

Variable	Patients with gastroesophageal reflux disease *n* = 21,486		Comparison patients *n* = 21,486		*p* value
	Total no.	Column %	Total no.	Column %	
Male	9812	45.7	9812	45.7	>0.999
Age, mean, SD (years)	53.0 ± 15.4		52.9 ± 15.9		0.308
Urbanization level					<0.001
1 (most urbanized)	7388	34.4	6878	32.0	
2	6116	28.5	5868	27.3	
3	3202	14.9	3530	16.4	
4	2674	12.4	2814	13.1	
5 (least urbanized)	2106	9.8	2396	11.2	
Geographic region					<0.001
Northern	10,670	49.6	10,222	47.6	
Central	5670	26.4	4976	23.2	
Southern	4678	21.8	5842	27.2	
Eastern	468	2.2	446	2.0	
Monthly income					<0.001
NT$0~15,840	7626	35.5	8020	37.3	
NT$15,841~25,000	7728	36.0	7728	36.0	
≥NT$25,001	6132	28.5	5738	26.7	

Note: The exchange rate in 2010 was US$1.00 ≈ New Taiwan (NT)$30.

Table 2 presents the mean annual ARI episodes per patient during the year before and after the index date among the study group and comparison group. GERD patients’ mean ARI episode rate decreased from 4.39 during the year prior to the index date to 3.89 after the index date (mean change = -0.5, 95% confidence interval (CI) = (-0.64, -0.36)). Among comparison patients, we observed a slight increase in the annual ARI episode rate after the index date (mean change = 0.08, 95% CI = (0.01, 0.15)). Fig 1 shows the distribution of patients by their annual ARI episode rate in the before-after index date years, classified by patient group. The graph shows a reduction in the annual ARI episode rate among GERD patients. In contrast, among the comparison patients, the ARI episode rate was similar in the two periods.

**Table 2:** Number of annual episodes of acute respiratory infection per patient during the year before and after the index date, among study patients (with gastroesophageal reflux disease, GERD) and comparison patients.

	Before		After		Difference (After-Before)	Paired *t*-test *p* value
	Mean annual ARI episodes	SD	Mean annual ARI episodes	SD	Mean (95% CI)	
GERD patients who received treatment	4.39	7.50	3.89	7.17	-0.50 (-0.64~ -0.36)	<0.001
Comparison patients	2.38	3.83	2.47	4.10	0.08 (0.01~0.15)	0.021

SD, standard deviation.

CI, confidence interval.

Table 3 shows the results of Poisson regression analysis using the difference-in-difference method. The coefficient of the interaction term between GERD treatment and time period was statistically significant and negative, after adjusting for patient’s age, sex, monthly income, urbanization level, and geographic region (estimate = -0.156, 95%CI = (-0.177, -0.134)).

**Table 3:** Poisson regression analysis, difference-in-difference method: Impact of receiving treatment for gastroesophageal reflux disease (GERD) on the annual number of acute respiratory infection episodes.

	Dependent variable: Total Number of acute respiratory infection episodes			
	Parameter estimate	Standard error	95% CI	P value
GERD treatment	0.614	0.008	0.598~0.629	<0.001
Time period (after vs. before)	0.035	0.009	0.017~0.052	<0.001
GERD treatment x Time period	-0.156	0.011	-0.177~ -0.134	<0.001
Age	-0.003	0.000	-0.003~ -0.002	<0.001
Male	-0.137	0.006	-0.154~ -0.133	<0.001
Urbanization level	-0.001	0.002	-0.005~0.004	0.821
Geographic region	0.035	0.003	0.029~0.041	<0.001
Monthly income	0.019	0.003	0.013~0.026	<0.001

Note: The exchange rate in 2010 was US$1.00 ≈ New Taiwan (NT)$30.

## Discussion

Based on reviewing the documented literature, this is the first study to examine the benefit of treating GERD with a PPI in reducing the ARI complications of GERD. Specifically, while a slight increase of 0.08 ARI episodes per year was observed among patients without GERD, the annual ARI rate among GERD patients dropped significantly, by 11.4%, from a mean of 4.39 episodes during the year before treatment to 3.89 after treatment with a PPI. This reduction is substantial, considering that ARI was ranked among the top five contributors to healthcare spending in Taiwan in 2014 (approximately NT$20 billion). The univariate findings are corroborated by multivariate Poisson regression analysis, which showed that GERD treatment was independently associated with a significant reduction in ARI episodes compared to the same patients’ rate before treatment and to the demographically comparable general population sample.

GERD is a common disease that causes coexisting or non-concurrent esophageal and extra-esophageal symptoms. Respiratory symptoms associated with GERD are some of the most widely prevalent and challenging extra-esophageal manifestations. For instance, GERD is the third leading cause of cough, affecting an estimated 20% of GERD patients [16,17]. GERD-related coughs mostly occur after an upper respiratory tract infection [18]. There is increasing epidemiological evidence of a strong relationship between reflux episodes and respiratory symptoms [10,12]. In a study that recruited 515 patients presenting to gastroenterology clinics in Saudi Arabia [12], a significant difference was observed in the prevalence of all respiratory symtpoms among patients with abnormal endoscopic findings (63%), compared to 37.2% among those with normal endoscopy. A positive correlation between the severity of airway obstruction as detected by FEV_1_ and FEV_1_/FVC and GERD symptoms was also observed.

GERD patients with extra-esophageal manifestations or complications, including respiratory diseases, have significantly higher healthcare utilization with associated higher costs [13]. These findings highlight the need for specific evidence on the impact of GERD treatment on non-esophageal complications. Acid-suppressive therapy, especially with PPIs, is generally the first-line approach for treating GERD. The efficacy of PPI therapy in relieving heartburn symptoms and healing erosive esophagitis [19,20], is validated by normal endoscopy findings among most patients with GERD following PPI treatment [4,21]. However, GERD treatment has not been impactful in resolving chronic symptoms of cough, hoarseness, and globus [22,23]. In a review of 12 randomized-controlled trials, a null finding was reported regarding overall symptom improvement in asthma patients following the medical treatment of GERD [24]. Our study presents a new finding regarding the impact of GERD treatment with PPIs on ARI episode rates, with the potential to reduce healthcare utilizaiton on this account.

There are several plausible reasons for fewer ambulatory visits for ARI following GERD treatment. GERD is recognized as a plausible cause of respiratory symptoms [25]. This may be due to the direct noxious effects of gastric contents on the mucosal surfaces of the upper airways (pharynx, larynx, middle ear, and nasosinusal complex) and lower airways (tracheobronchopulmonary tree). Lacking protection from the antireflux clearance mechanisms and intrinsic mucosal properties present in the lower esophagus, the airways may be more readily damaged and functionally impaired by the reflux of gastric contents beyond the esophagus. Persisting reflux episodes, often produced by the transient relaxation of the lower esophageal sphincter, causing reflux contents to spread beyond the esophagus into the airways could plausibly cause respiratory symptoms [12,26]. Acid-suppressive medication reduce reflux episodes, and may allow the airways to heal, as well as restore their normal anatomic and functional mechanisms due to stopping the stimulation and damage by regurgitated gastric juices. These effects may clinically manifest as a reduction in ARI episodes, in turn reflected in reduced ambulatory care visits for ARIs, the measure that we used in this study. A second biological, direct effect of PPI may be activated: the known attenuation of neutrophil adherence to endothelial cells by PPIs via inhibiting the expression of adhesion molecules, which is thought to exert an anti-inflammatory effect [27]. Because inflammatory mediators in the airways are elevated in many chronic respiratory conditions such as post-nasal drip, asthma and GERD [28], it is plausible that PPI treatment of GERD initiates an anti-inflammatory process that reduces airway inflammation and reduces the risk of airway infections. It is documented that even a single reflux episode extending beyond the esophagus may be sufficient to trigger respiratory signs and symptoms [12].

One limitation of our study is that the duration of GERD symptoms before seeking medical treatment was not available. However, this limitation would result in underestimation of the GERD treatment effect, as the study patients’ initial reflux episodes most likely, started less than a year before the GERD diagnosis was made (which allowed them to be included in the study; patients with pre-exisiting GERD diagnosis were excluded from the study). Comparing their ARI healthcare utilization during the year before and after GERD treatment would have underestimated the benefits of GERD medication on ARIs. Despite this potential effect, our study finds a measurable and significant reduction in ARI episodes following GERD treatment with PPIs.

There are significant implications of the study which highlights the importance of educating patients to seek medical treatment for GERD syndromes. GERD is defined as “a condition which develops when the reflux of stomach contents causes troublesome symptoms and/or complications” [1]. The patient-centered approach emphasizes the imperative to increase GERD symptom awareness among the general population and to encourage patients to seek prompt medical treatment. The population should be educated that once the symptoms of heartburn and regurgitation impact their regular activities of living and quality of life, timely treatment is important to relieve symptoms and minimize the complications, including ARIs. Studies suggest that PPI compliance is generally poor [29], with about 40%~50% of patients not taking the prescribed PPIs correctly [30,31]. It is also documented that both healthcare resource use and costs were reduced among PPI compliants compared to their pre-PPI period, and compared to noncompliant patients [32]. Patient education needs to be reinforced for successful PPI treatment.

A particular strength of this study was the use of a longitudinal, nationwide, population-based sample cohort, with comprehensive follow-up data due to mandatory enrollment in Taiwan’s NHI program. Selection bias may, therefore, be minimal. Another strength is that the GERD diagnosis was endoscopy-confirmed, and additionally validated by an independent expert sub-committee (constituted to approve PPI use). We also limited the study criteria to only include patients with a substantial duration of GERD treatment (claim for a PPI for more than 30 days). These inclusion criteria protect against misclassification bias and ensure clear separation of study and comparison patients on the index diagnosis.

There were some study limitations. One inherent limitation of using claims data is that the study gets limited to pateints who sought treatment for GERD and ARIs. Due to the wide range of GERD manifestations and subjective preferences for action-taking and symptom perceptions, the real prevalence of pathological reflux disease is thought to be underestimated [33]. However, the relevance of this issue for our study findings may be mitigated. Individual behaviors of healthcare utilization may be fairly consistent over time. Pre- vs. post-treatment comparisons among the same groups of patients, with and without GERD should therefore attenuate this source of concern. Second, the study data do not permit studies of healthcare cost differences due to GERD treatment because claims data do not have information on GERD severity. Aggregating claims costs without accounting for severity would bias the results. Third, our estimates of healthcare utilization may be biased due to not accounting for over-the-counter medications used for GERD. This may be mitigated by the fact that Taiwan has a universal coverage, National Health Insurance program since 1995, with very low out-of-pocket payments which facilitates patient access to providers. Further, the high cost of PPI medications may discourage over-the-counter purchase. Another limitation is the lack of data on medication compliance, a documented phenomenon with PPI medication. However, sub-optimal compliance should bias the findings towards the null. Another limitation is that despite a finding of reduced ARI rates following PPI use, it does not mean that the PPI treatment was adequate. It is possible that optimal GERD treatment may reduce the ARI episode rate further on a population-basis. On a precautionary note, assessing the magnitude of ARI reduction should be in the context of the benefits and risks of PPI treatment (e.g., side effects and complications). Finally, again due to the limitations of claims data, our study was unable to account for other important risk factors such as the body-mass index, cigarette smoking, alcohol consumption, other medications being used, and dietary habits (e.g., soda and coffee consumption) that may impact both GERD and ARIs [34,35]. Two study features mitigate this concern. While data on co-morbidities and other medications were unavailable, the random selection of age-sex matched comparison patients may have resulted in a reasonably representative sample of the non-GERD general population. Further, comorbidities, other medications, and life style factors within the same individual may be stable over a 2-year period, and less critical to adjust for, given our use of a pre- vs. post-treatment comparison approach.

GERD has increasingly been identified as a potential cause of respiratory symptoms, translating into substantial clinical and economic burden for healthcare systems. This large-scale population-based cohort study suggests that there are potential benefits of GERD treatment in reducing healthcare visits for ARIs. More studies, particularly stratifying patients by the nature and severity of both ARI and GERD symptoms, are needed to validate our findings and produce more robust evidence for clinical practice and policy changes. Cost effectiveness, cost-benefit and quality of life studies are also essential to evaluate the full impact of PPI treatment on patients’ health, quality of life, and healthcare expenditures.
